# Cardiac tamponade mimicking tuberculous pericarditis as the initial presentation of chronic lymphocytic leukemia in a 58-year-old woman: a case report

**DOI:** 10.1186/1752-1947-4-246

**Published:** 2010-08-04

**Authors:** Elaine Lin, Adrienne Boire, Vagish Hemmige, Aliya N Husain, Matthew Sorrentino, Sandeep Nathan, Shahab A Akhter, Jerome Dickstein, Stephen L Archer

**Affiliations:** 1 Pritzker School of Medicine, University of Chicago, Chicago, IL, USA; 2 Department of Medicine, University of Chicago, Chicago, IL, USA; 3 Department of Pathology, University of Chicago, Chicago, IL, USA; 4 Section of Cardiology, Department of Medicine, University of Chicago, Chicago, IL, USA

## Abstract

**Introduction:**

Chronic lymphocytic leukemia is an indolent disease that often presents with complaints of lymphadenopathy or is detected as an incidental laboratory finding. It is rarely considered in the differential diagnosis of patients presenting with tamponade or a large, bloody pericardial effusion. In patients without known cancer, a large, bloody pericardial effusion raises the possibility of tuberculosis, particularly in patients from endemic areas. However, the signs, symptoms and laboratory findings of pericarditis related to chronic lymphocytic leukemia can mimic tuberculosis.

**Case Presentation:**

We report the case of a 58-year-old African American-Nigerian woman with a history of travel to Nigeria and a positive tuberculin skin test who presented with cardiac tamponade. She had a mild fever, lymphocytosis and a bloody pericardial effusion, but cultures and stains were negative for acid-fast bacteria. Assessment of blood by flow cytometry and pericardial biopsy by immunohistochemistry revealed CD5 (+) and CD20 (+) lymphocytes in both tissues, demonstrating this to be an unusual manifestation of early stage chronic lymphocytic leukemia.

**Conclusion:**

Although most malignancies that involve the pericardium clinically manifest elsewhere before presenting with tamponade, this case illustrates the potential for early stage chronic lymphocytic leukemia to present as a large pericardial effusion with tamponade. Moreover, the presentation mimicked tuberculosis. This case also demonstrates that it is possible to treat chronic lymphocytic leukemia-related pericardial tamponade by removal of the fluid without chemotherapy.

## Introduction

Chronic lymphocytic leukemia (CLL) is an indolent disease that often presents with complaints of lymphadenopathy and fatigue or is detected as an incidental laboratory finding. Although leukemias represent only about eight percent of neoplastic metastases to the heart, almost 50% of lymphoma patients have cardiac involvement at autopsy [1]. Pericardial effusions or cardiac tamponade are relatively uncommon as presenting syndromes in patients with hematologic malignancies [2,3]. Likewise, hematologic malignancies account for a minority of pericardial effusions. Imazio *et al *found that only 33 (7.3%) of 450 patients with acute pericardial disease had a neoplastic etiology. The most powerful clinical predictor of a neoplastic etiology was a history of malignancy (odds ratio 19.8) [4]. Lung and breast cancers are the most common neoplasms causing pericardial effusion [4]. In a series of 150 cases of cardiac tamponade requiring pericardiocentesis, 64% had sanguinous pericardial fluid. The most common causes of the effusions were iatrogenic (31%), followed by malignancy (26%) [5]. Only a handful of cases have described cardiac involvement in CLL and these reports described patients with established CLL, rather than patients presenting with tamponade and being subsequently discovered to have CLL [2,6-8]. We present the case of a woman whose initial presentation of CLL was cardiac tamponade with sanguinous pericardial fluid but whose history and clinical presentation was suspicious for tuberculous pericarditis.

## Case presentation

A 58-year-old African American-Nigerian woman with a week-long history of progressive shortness of breath presented to our emergency room. She had lived in Benin, Nigeria until nine years earlier and had been back for a one-month-long visit three years prior to presentation. She had previously been in excellent health until one week prior to admission. Over the week she became dyspneic with minor exertion and could no longer climb one flight of stairs without pausing to rest. She denied any chest pain, but described a sense of 'congestion' in her chest, which progressed over the week. The patient had a subjective fever that began at the same time as the dyspnea and which was relieved by acetaminophen. Her past medical history was significant for hypertension (treated with hydrochlorothiazide and amlodipine) and a positive tuberculin skin test.

On physical examination, her vital signs included a heart rate of 100 and blood pressure of 134/94 with a pulsus paradoxus of over 15 mm Hg. Her cardiovascular examination revealed distant heart sounds, normal first and second heart sounds and no murmurs, rubs or knocks. There was jugular venous distention to 20 cm above the manubriosternal angle. The lung examination was unremarkable. Her abdomen was soft and nondistended. There was no peripheral edema, adenopathy or hepatosplemogaly.

An initial laboratory assessment was notable for leukocytosis and elevated liver enzymes (Table 1). The chest x-ray revealed cardiomegaly and a small left pleural effusion. The electrocardiogram showed mild QTc prolongation (610 ms) but no signs of low-voltage, electrical alternans or ischemia. The D-dimer level was elevated at 3.16 mg/dl. A multislice computed topography scan, performed to exclude pulmonary embolism, showed no pulmonary embolism, but did reveal mediastinal lymphadenopathy and a large pericardial effusion. Given the her immigrant history and past positive PPD, a working diagnosis of tuberculosis pericarditis was entertained, and she was placed on respiratory isolation.

**Table 1:** Initial Lab Values

White Blood Cell (3.5 to 11 k/uL)	18.1
Red Blood Cell (3.88 to 5.26 M/uL)	3.59
Absolute Lymphocytes (0.9 to 3.3 K/uL)	10.50
Absolute Reactive Lymphocytes	0.54
Absolute Monocytes (0.16 to 0.92 K/uL)	1.63
Hemoglobin (11.5 to 15.5 g/dL)	11.1
Hematocrit (36 to 47%)	33.3
Sodium (134 to 149 mEq/L)	138
Potassium (3.3 to 4.7 mEq/L)	3.2
Chloride (95 to 108 mEq/L)	100
Carbon Dioxide (23 to 30 mEq/L)	23
Blood Urea Nitrogen (7 to 20 mg/dL)	12
Creatinine (0.5 to 1.4 mg/dL)	0.8
SGOT (8 to 37 U/L)	168
SGPT (8 to 35 U/L)	226
D-Dimer Assay (<0.42 ug/ml)	3.16
Ferritin (10 to 220 ng/mL)	601
ANA titer (0 to 80)	160
Anti-ds DNA (<10 titer)	<10

**Figure 1:**
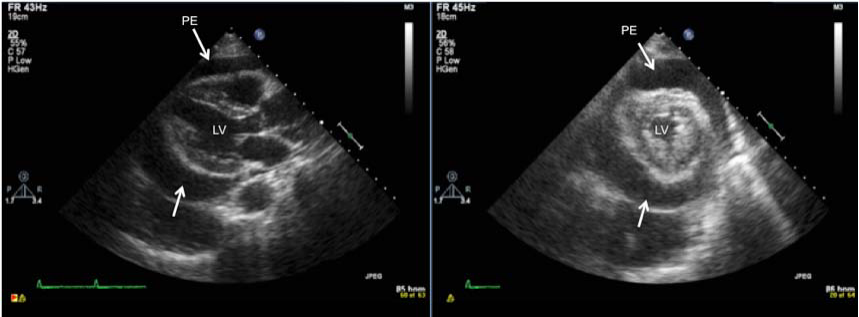
**A transthoracic 2-dimensional echocardiogram**. Note the circumferential pericardial effusion on the long- and short-axis parasternal views on this transthoracic echocardiogram, prior to pericardiocentesis. PE, pericardial effusion; LV, left ventricle

A subsequent transthoracic echocardiogram (Figure 1) showed a large, circumferential pericardial effusion with evidence of right ventricular collapse. Though the her blood pressure remained normal, her jugular venous distention and pulsus paradoxus were consistent with incipient tamponade and pericardiocentesis was performed. Approximately 1L of sanguinous fluid was extracted from the pericardial sac. With drainage the patient immediately improved and had resolution of her dyspnea and normalization of her physical examination.

The pericardial fluid contained 2,680,000 red blood cells and 8,500 leukocytes/uL. The leukocyte differential was 33% neutrophils, 56% lymphocytes, 6% macrophages, 2% mesothelial cells and 3% eosinophils. Fluid analysis showed that the fluid glucose was 59 mg/dl, fluid lactate dehydrogenase was 325 IU/L, and total protein was 4.5 g/dl. Staining of the fluid for acid-fast bacilli was negative, as were bacterial cultures. Fluid cytology revealed only reactive mesothelial cells.

Because of persistent concern about possible tuberculous pericarditis and lack of a definitive diagnosis, a pericardial window was performed from a subxiphoid approach. On gross examination, the thickened pericardium measured between 0.1 and 0.3 cm. However, acid-fast stains and culture remained negative.

On the sixth day after admission, the daily complete blood count with differential was notable for the presence of immunoblasts. Flow cytometry of peripheral blood for lymphocyte subsets was performed. A repeat echocardiogram did not demonstrate reaccumulation of pericardial fluid and the patient remained asymptomatic and was discharged home for outpatient evaluation.

**Figure 2:**
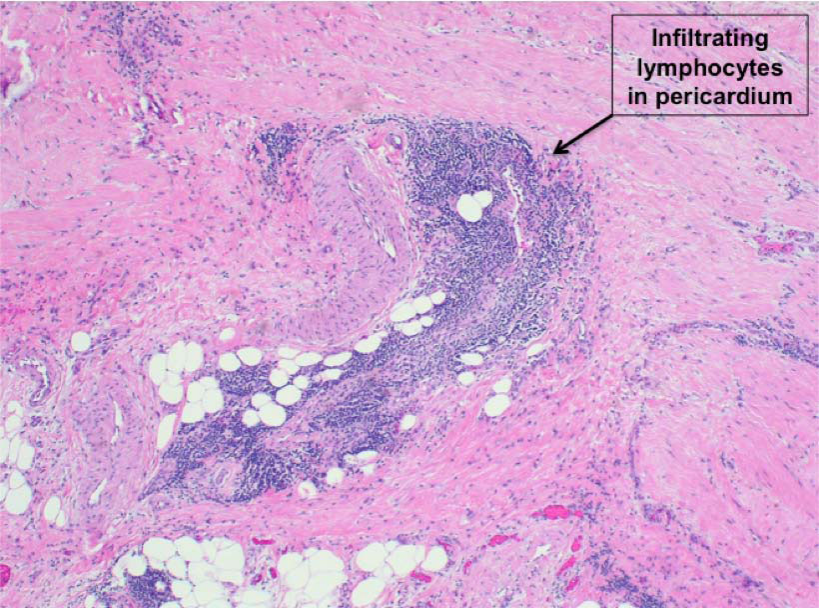
**A pericardial biopsy (Hematoxylin and eosin stain)**. Note the focal lymphocytic infiltrate in the pericardial tissue removed at the time of the subxiphoid pericardial window.

**Figure 3:**
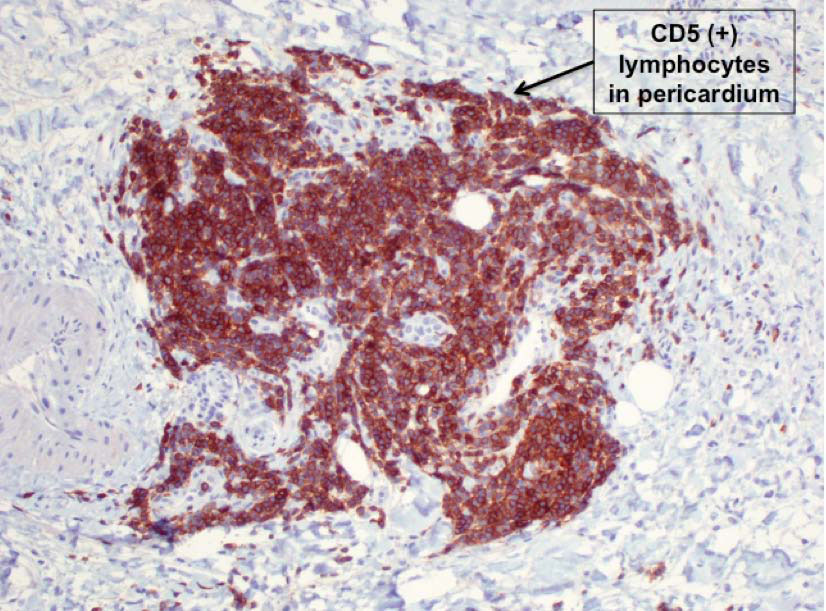
**A pericardial biopsy (immunohistochemical stain)**. CD5 stain of pericardial tissue demonstrated that the vast majority of the lymphocytes were CD5 positive (brown stain).

The flow cytometry results were consistent with CLL. The B/T ratio was 1.8:1. B-cells expressed CD5, CD19, CD20, CD21 (partial), CD22, CD23, CD11c (partial) and CD52, consistent with CLL. Subsequently, histological examination of the pericardium revealed lymphocytic infiltrates surrounding the vascular structures and dispersed within the adipose tissue (Figure 2). The infiltrate was comprised of small lymphocytes with clumped chromatin, indistinct nucleoli and high nuclear/cytoplasmic ratio. Immunohistochemistry of the pericardial tissue demonstrated that the vast majority of the lymphocytes were CD5 (+) (Figure 3) and CD20 (+) B-cells, with a minority of lymphocytes being CD3 (+) T-cells. Together, the flow cytometry of the peripheral blood and immunohistochemistry of the pericardial tissue were consistent with pericardial involvement by CLL.

The patient was seen in follow-up at one year and has remained free from symptomatic disease without any chemotherapy. Echocardiography demonstrated that her pericardial effusion had not recurred.

## Discussion

The etiologies of sanguinous pericardial effusions causing tamponade, excluding iatrogenic causes, include malignancy, renal failure and/or uremia and tuberculosis, although the latter is now uncommon in North America [5]. Our initial high suspicion for tuberculous pericarditis was based on her history, the bloody fluid, the mediastinal adenopathy, and the lymphocytosis in the pericardial fluid. An adenosine deaminase assay, which has been used to identify tuberculosis, was uninterpretable due to the amount of blood in the fluid [9]. Initially, we discharged the patient under the assumption that the tamponade was most likely due to a viral cause, although Coxsackie and adenoviral titers were negative. It was the late appearance of circulating immunoblasts that finally pointed towards a leukemic process.

Up to 20% of patients with a known malignancy are found to have pericardial involvement upon autopsy [10]. However, tamponade as an initial manifestation of malignancy is relatively uncommon. A review of 78 cases revealed that 60% of such cases stemmed from lung carcinomas whereas only 9% originated from leukemia or lymphoma [11]. The evolution of pericardial effusion is often due to infiltration of malignant cells as well as lymphatic obstruction; our patient clearly had pericardial involvement of leukemia on histology.

A PubMed search beginning in 1979 using the keywords "chronic lymphocytic leukemia and pericarditis, pericardial tamponade, pericardial effusion" identified only a handful of cases that have documented cardiac infiltration in patients with CLL. One case describes tamponade and pericardial effusion as the initial presentation of lymphosarcoma cell leukemia, which is morphologically very similar to CLL [12]. This patient presented with dyspnea and mild abdominal distention and had a leukoctye count of 23,200/uL. Three other cases described tamponade related to previously documented CLL in patients presenting with dyspnea [3,7,8]. Finally, one case report detailed constrictive pericarditis in a patient with B-cell chronic lymphatic leukemia whose initial complaint was also breathlessness [6]. The leukocyte count in all the CLL patients in these reports was much higher (282,000 to 827,000/uL) than our patient's (18,100/uL).

She subsequently continued follow-up with a primary oncologist regarding the status of her CLL. She was found to be Rai stage I and one-year post discharge had not received CLL treatment (because she had normal platelet and hemoglobin levels and remained asymptomatic). Management of pericardial effusions as initial presentations of malignancy is not well established, though some reports have suggested systemic chemotherapy and radiotherapy prior to pericardiocentesis to avoid potential complications [13]. In our patient, because the diagnosis of leukemia was unknown, pericardiocentesis was performed without chemotherapy and has provided sustained relief of her dyspnea for the past year.

## Conclusion

To the best of our knowledge this is the first reported case of CLL presenting as pericardial tamponade. The diagnosis was confounded by the similarities to tuberculous pericarditis and the modest degree of leukocytosis. The appearance of peripheral immunoblasts was the key to the ultimate diagnosis, which we confirmed by demonstrating CD5 (+) and CD20 (+) lymphocytes using flow cytometry on the blood and immunohistochemistry on the pericardial tissue.

## Competing interests

The authors declare that they have no competing interests.

## Consent

Written informed consent was obtained from the patient for publication of this case report and accompanying images. A copy of the written consent is available for review by the Editor-in-Chief of this journal.

## Authors' contributions

EL and SLA were involved in the conception, design, drafting, and revising of the manuscript. All authors were involved in the diagnosis and treatment of the patient and revising the manuscript. All authors read and approved the final manuscript.
